# Retraction Note: Downregulation of RKIP promotes radioresistance of nasopharyngeal carcinoma by activating NRF2/ NQO1 axis via downregulating miR-450b-5p

**DOI:** 10.1038/s41419-023-05861-6

**Published:** 2023-05-31

**Authors:** Wei Huang, Guangqing Shi, Zhong Yong, Jian Li, Juan Qiu, Yan Cao, Yongfeng Zhao, Li Yuan

**Affiliations:** 1 Department of Nuclear Medicine, The Third Xiangya Hospital, Central South University, Changsha, Hunan 410013 China; 2 Research Center of Carcinogenesis and Targeted Therapy, Xiangya Hospital, Central South University, Changsha, Hunan 410008 China; 3 Department of Ultrasound, the Third Xiangya Hospital, Central South University, Changsha, Hunan 410013 China

Retraction to: *Cell Death and Disease* 10.1038/s41419-020-2695-6, published online 06 July 2020

The Editors-in-Chief have retracted this article at the request of the corresponding author. Concerns were raised regarding a number of figures, specifically:

- Figure 2d: the TUNEL shNC panel for CNE2 appears to partially overlap with the yH2AX shRKIP+shNRF2 panel for CNE2
- Figure 2: the TUNEL shRKIP panel for CNE2 appears to partially overlap with the yH2AX shRKIP panel for CNE2
- Figure 6a: the NRF2 Radioresistant NPC panel appears to be partially overlap with the phosphoStat3 panel in Figure 1A in a previously published article by different authors [1]

The Editors-in-Chief therefore no longer have confidence in the results and conclusions of this article.

The corresponding author has stated on behalf of all authors that they agree with this retraction.

